# Supplementary material for: Distributary development in a 21st century river: The evolution of Neptune Pass and its delta, the largest new offshoot of the Mississippi River
Source: PLoS One. 2025 Apr 16;20(4):e0320502. doi: 10.1371/journal.pone.0320502 (PMC12002485; doi:10.1371/journal.pone.0320502)
Supplement: S1 Supplemental Material — Data from 5/24/2022 and 2/16/2022 are from these authors while the other data are from the US Army Corps of Engineers routine surveys and made available by distribution from Eden Krolopp and colleagues. In some cases, surveys were conducted over 2-day period, and here the first day of that survey is listed). The units for discharges are m3 s-1. (DOCX) [file pone.0320502.s001.docx]

Supplemental Material 1

Discharge Data In The Mississippi River. Data from 5/24/2022 and 2/16/2022 are from these authors while the other data are from the US Army Corps of Engineers routine surveys, and made available by distribution from Eden Krolopp and colleagues.

In some cases, surveys were conducted over 2-day period, and here the first day of that survey is listed).

The units for discharges are m^3^ s^-1^.

| Date | Belle Chasse | Above Ostrica | Neptune Pass | Olga Upsteam | Olga Downstream | Water Loss, Belle Chasse To Olga Downstream | Water Loss Vs Above Ostrica | % Discharge, Neptune Pass Vs Belle Chasse | % Water Loss, Belle Chasse To Olga Downstream | %Water Loss Vs Above Ostrica |
| --- | --- | --- | --- | --- | --- | --- | --- | --- | --- | --- |
| 5/24/22 | 22,083 | 20,290 | 3,358 |  | 12,322 | 9,761 | 7968 | 15.206 | 36.1 | 39.3 |
| 8/23/22 | 9,789 | 10,727 | 1,685 | 9,472 | 9,346 | 443 | 1381 | 17.214 | 14.1 | 12.9 |
| 4/4/23 | 22,254 | 21,615 | 3,443 | 19,054 | 15,636 | 6,618 | 5979 | 15.47 | 26.9 | 27.7 |
| 4/17/23 | 25,726 | 23,827 | 3,633 | 20,893 | 17,450 | 8,275 | 6376 | 14.124 | 24.8 | 26.8 |
| 4/18/23 | 25,100 |  |  | 20,893 | 17,450 | 7,649 |  |  | 0 |  |
| 5/15/23 | 14,912 | 15,148 | 2,195 | 12,980 | 10,922 | 3,989 | 4226 | 14.718 | 28.3 | 27.9 |
| 6/12/23 | 9,021 | 8,976 | 1,875 | 7,751 | 6,130 | 2,891 | 2846 | 20.784 | 31.5 | 31.7 |
| 7/25/23 | 7,513 | 7,925 | 1,358 | 7,117 | 6,080 | 1,432 | 1845 | 18.075 | 24.6 | 23.3 |
| 9/19/23 | 3,728 | 5,219 | 948 | 3,982 | 2,356 | 1,372 | 2864 | 25.432 | 76.8 | 54.9 |
| 1/30/24 | 17,786 | 15,835 | 2,065 | 14,476 | 11,297 | 6,489 | 4537 | 11.611 | 25.5 | 28.7 |
| 2/16/24 | 22,055 | 20,531 | 3,205 |  | 14,142 | 7,913 | 6389 | 14.532 | 29 | 31.1 |
| 2/27/24 | 17,359 | 16,121 | 2,714 |  | 11,255 | 6,104 | 4866 | 15.636 | 28 | 30.2 |
| 8/23/22 | 9,789 | 10,727 | 1,685 | 9,472 | 9,346 | 443 | 1381 | 17.214 | 14.1 | 12.9 |
