# Supplementary material for: Distributary development in a 21st century river: The evolution of Neptune Pass and its delta, the largest new offshoot of the Mississippi River
Source: PLoS One. 2025 Apr 16;20(4):e0320502. doi: 10.1371/journal.pone.0320502 (PMC12002485; doi:10.1371/journal.pone.0320502)
Supplement: Attachment — Submitted filename: Response_To_Reviewrs_Dec_5.docx [file pone.0320502.s003.docx]

Dear PLOS One:

Please see this file that contains a response to the comments provided by the reviewer, editor, and journal. We believe the comments these individuals provided greatly improved the quality of the manuscript, and we hope that it now meets PLOS One's standards for publication.

Should you have further questions, please do not hesitate to ask me.

My co-authors and I look forward to working with PLOS One.

Sincerely,

-Alexander S. Kolker

Response To Reviewer:

Reviewer #1: Excellent paper that has left no ground unturned. Will be useful to both delta geoscientists and delta managers. Well done!

*> Thank you!*

Minor points to rectify:

Line 118: potentially leading to ...

*Done. Please see line 119 in the tracked changes version.*

Lines 122ff and 126ff: Where do these hypotheses come from? If you are presenting them here for the first time then reword this as the presentation looks awkward and gives the impression that that these hypotheses are already extant.

*This has been addressed. Please see lines 124- 125 in the tracked changes version.*

Line 145: "to generate (rather than provide in order to avoid using "provide" twice in the same sentence).

*This has been addressed. Please see line 151 in the tracked changes version.*

Line 146ff: This information should also be useful to delta geoscientists, so although this is mentioned above, it can be reiterated following the management bit for delta geoscience management elsewhere.

*Yes. We agree that this information should be useful to a broad range of geoscientists. We now note this in lines 152-153 of the tracked changes manuscript.*

Line 809: Fig. 14. Need to specify that this delta cycle refers to the Mississippi and reference Roberts as reference 18.

*This has been addressed. Please see line 873 of the tracked changes version.*

Line 584: "also consistent with ..."

*This has been addressed (We believe the reviewer was actually referring to line 846)*

Please see line 911-912 of the tracked changes version.

Figures: Figure 1 is a bit disappointing. For an international readership, please show a regional USA map with the Mississippi delta in inset and add geographical coordinates to the figure.

*Thank you for this comment. We have fixed Figure 1 following the suggestions of this reviewer. We hope it now satisfies this reviewer.*

Response to Editor's Comments:

Editor Comment 1. The study primarily relies on conventional methods for analyzing sediment transport and land formation, such as sediment core collections and remote sensing, which may not capture more complex aspects of distributary development and delta dynamics.

*> We thank the editor for thoughtful comment, and we appreciate the editor's desire to understand the complex aspects of distributary development. However, we used the methods we used because they work. We think scientific methods should be judged based on whether they are appropriate to address specific questions at hand, rather than whether they are novel.*

*We appreciate the editor's interested in seeing new methods. We turn their attention to satellite-derived bathymetry, and the use high-resolution LIDAR (ie. Sections 3.6 and 3.6 and Figs 7, 8 and 12. We now highlight how this study pioneers the use satellite-derived bathymetry in a river delta. See lines 937-940 of the tracked changes version.*

*Our study also advances the technology of LIDAR DEM+RBG and reflectivity in river deltas Section 3.5 and Fig 7). We now highlight how this technology can be used in river deltas, where it's use has previously been limited. Please see lines 650-652 of the tracked changes version.*

Editor Comment 2.

Additionally, while it investigates sediment dynamics in Neptune Pass, it lacks a deeper exploration of the broader environmental or hydrological impacts, missing a nuanced understanding of how these processes affect local and regional ecosystems.

*> While we very much appreciate the editor's desires to see a discussion of the broad hydrological impacts of Neptune Pass, we believe the comment that our study lacks this material is incorrect. We dedicate a significant amount of text to describing Neptune Pass's broader environmental impacts. Key examples of places where describe the environmental impacts of the system include:*

*Section 5.1: Presents a detailed discussion of the local, regional and global implications of Neptune Pass. (Lines 729 - 777 of the tracked changes manuscript)*

*Section 5.3.1, and 5.3.2 Presents a description of the development of Neptune Pass and the Quarantine Bay Delta in the context of geomorphic theory. (Lines 864 - 959 of the tracked changes manuscript)*

*Section 5.3.3. Presents a discussion of development of deltas in the context of local vegetation. (Lines 965-986 of the tracked changes manuscript)*

*Section 5.4. and 5.5. Presents a detailed discussion of the development of Neptune Pass and the Quarantine Bay Delta in the context of regional environmental planning. (Lines 1014-132, and 1034-1109 of the tracked changes manuscript)*

Editor Comment 3

The results are presented with varying levels of detail, which complicates full interpretation, as sediment volumes are reported with different levels of confidence and assumptions, potentially affecting the reliability of the conclusions.

*> We appreciate the reviewer's desire to see the results with a more specific level of detail. We clarify here that both the scour estimates and deposition estimates are guided by marine sonars, and thus have a strong degree of comparability. To further clarify how the sediment volumes were calculated, we added an additional sentence that reads, "Using differing values for the porosity of sediments in Neptune Pass and Quarantine Bay provides a means of bracketing the range of scenarios for deposition and erosion."* See lines 712-714 of the tracked changes version.

Editor Comment 4

The discussion on potential management strategies is somewhat generic, lacking specific recommendations or actionable insights that could directly inform policy or management practices.

*> We thank the editor for asking to see additional material on management. To address this comment, we clarified and expanded Section 5.5, which examines the management implications of this system, and pathways for future management related research. Please see the entirety of Section 5.5, lines 1034-1109 of the tracked changes version.*

Moreover, the paper does not adequately compare its findings with other studies or historical data, which would help contextualize the significance of Neptune Pass’s development within a broader framework of delta evolution and river management.

*> While we very much appreciate the editor's desire to see historical data and comparisons to other systems, this material is already in the paper. Please see:
Section 5. 1 Neptune Pass Discharge in Perspective:*

**We compare the size of Neptune Pass to other crevasses/river locally, nationally and globally. Please see lines 729-744 of the tracked changes version.*

** We compare the size of Neptune Pass to one of the largest environmental restoration projects in the United States, the Mid Barataria Sediment Diversion. Please see lines 746-760 of the tracked changes version.*

*Section 5.3 Development of Neptune Pass in the context of deltaic development.*

*Section 5.3.1. The delta cycle.*

**We compare Neptune Pass to well established theory on delta development.*

*Please see lines 863-908 of the tracked changes version.*

** We compare Neptune Pass to well established theory on the sediment dynamics at river mouths. Please see lines 911-969 of the tracked changes version.*

*5.3.3 The role of vegetation.*

** We compare Neptune Pass to well established ecogeomorphologic theory. Please see lines 965-986 of the tracked changes version.*

The study reports a net deposition of sediment in Quarantine Bay but does not fully account for possible variations in sediment density or other factors influencing the sediment budget, which could strengthen the findings.

*> The density of the sediments is not likely to vary significantly. Many siliciclastic sediments in the Mississippi River Delta, such as quartz (i.e. sand), feldspar, and clay minerals have a density that is close to 2600 kg/m3. such, many authors use this value in their calculations- see for example Blum and Roberts (2012)- citation 34. As such, we do not believe that any additional calculation is needed, or even appropriate.*

*However, there could be differences in the amount of pore space in the sediments. As such, we used two porosity scenarios in our sediment budget. The first calculation used a sediment porosity of 0.4- the value most commonly used in the literature [34]. In the second calculation we assumed the porosity of original sediments was less that typical (ie more tightly compacted that usual), and that the porosity of the deposited sediments was greater than typical (i.e. less tightly compact than usual) - using literature values. This provided a reasonable estimate of the range of porosities that could be exist in this system. We thus believe our methods specifically address this reviewer's concerns. This information is reflected in lines 501-513 of the tracked changes version.*

The discussion of the "new land" versus "redistributed sediment" hypotheses may oversimplify the sediment dynamics, as both processes might contribute to land development, warranting a more detailed examination of sediment sources and pathways.

*>We agree with the reviewer that both processes might contribute to land development, which is why we have specifically addressed this issue in the text. We refer the editor to lines 134 to 135 in the tracked changes version which reads, "(It should be noted that these hypotheses are not mutually exclusive and represent end-members on a spectrum of sediment input possibilities.". Please see lines 134-135 of the tracked changes version.*

*We also refer the editor to lines 1026-1032 which reads, "There are a few caveats to the aforementioned findings. First, the "new land" vs "redistributed land” hypotheses are not entirely mutually exclusive. It is theoretically possible, and even likely, that sediments deposited in Quarantine Bay could be derived from both channel scour and the Mississippi River......." Please see line 1026-1032 of the tracked changes version.*

Future predictions and management interventions are discussed without specific, data-driven scenarios; incorporating more detailed modeling and field studies could better predict the impacts of reduced flow or sediment retention measures on Neptune Pass.

*> We agree with the reviewer that more modelling studies are important. However, we believe that those are best left for future studies that build on this work. Following one of the editor's earlier remarks, we do now conclude the paper will a call for further research, and we hope that this call helps address the editor's concerns. Please see lines 1085-1093 of the tracked changes version.*

The generalizations about coastal management implications lack site-specific factors or challenges, and more precise recommendations tailored to the local context would be beneficial.

*>We have expanded the section on coastal management to include more site-specific material. We refer the editor to lines 1034-1108.*

Lastly, while the study highlights Neptune Pass as a net land-building system, it does not thoroughly explore the ecological consequences, such as impacts on local flora and fauna or changes in habitat conditions, which would provide a more comprehensive understanding of the delta's evolution.

*We thank the reviewer for their interest in the ecology of the region. We point out that In section 5.3.3, "The role of vegetation", our study describe the role of vegetation in Neptune Pass and its associated deltas. Our paper also refers the reader to the classical papers on ecogeomorphological theory for developing deltas in coastal Louisiana. While we would love to see more papers on the ecology of this system, those are best done by other manuscripts that could be submitted to this journal at another point in time. Please see lines 965-986 of the tracked changes version.*

Journal Comments

When submitting your revision, we need you to address these additional requirements.

1. Please ensure that your manuscript meets PLOS ONE's style requirements, including those for file naming. The PLOS ONE style templates can be found at

*> This has been addressed. To the best of our knowledge, this manuscript meets the standards of PLOS One.*

2. In your Methods section, please provide additional information regarding the permits you obtained for the work. Please ensure you have included the full name of the authority that approved the field site access and, if no permits were required, a brief statement explaining why.

*> We have added Section 3.9 Permitting Approval to the manuscript. This section describes the permits needed and provides details on the permits that were obtained.*

3. Please note that funding information should not appear in any section or other areas of your manuscript. We will only publish funding information present in the Funding Statement section of the online submission form. Please remove any funding-related text from the manuscript.

*> All funding related information has been removed from the manuscript. Of particular relevance- the material that was in section 6.0 Acknowledgements has been deleted so as to conform with journal policies.*

4. Thank you for stating the following financial disclosure: 
   "This project was funded, in part, through a sub-contract with the Louisiana Coastal Protection and Restoration Authority, who was funded under Award No. GNTCP18LA0035 from the Gulf Coast Ecosystem Restoration Council (RESTORE Council), and through multiple contracts with the National Wildlife Federation. This work represents contract #20220831Task Order No.4 from the Louisiana Coastal Protection And Restoration Authority to the Louisiana Universities Marine Consortium. The data, statements, findings, conclusions, and recommendations are those of the authors and do not necessarily reflect any determinations, views, or policies of the RESTORE Council. We acknowledge that Dr. Alisha Renfro from the National Wildlife Federation contributed to this manuscript in her capacity as a scientist and scholar. Links to project sponsors can be found here: <https://coastal.la.gov>; <https://www.restorethegulf.gov>; <https://www.nwf.org>"
 
Please state what role the funders took in the study.  If the funders had no role, please state: "The funders had no role in study design, data collection and analysis, decision to publish, or preparation of the manuscript." 
If this statement is not correct you must amend it as needed.  Please include this amended Role of Funder statement in your cover letter; we will change the online submission form on your behalf.

*> Thank you for asking for this clarification, our previous statement has been clarified. Our cover letter now includes the following text " We acknowledge that Dr. Alisha Renfro from the National Wildlife Federation contributed to this manuscript in her capacity as a scientist and scholar. In particular, some of her insights on the geology of the Mississippi River Delta were part of the discussion section. Louisiana's Coastal Protection And Restoration Authority, the project's other funder, had no role in study design, data collection and analysis, decision to publish, or preparation of the manuscript "*

5. In the online submission form, you indicated that "All data are available from the Louisiana Coastal Protection And Restoration Authority, by making a data request here:<https://cims.coastal.louisiana.gov/DataRequest.aspx>"

All PLOS journals now require all data underlying the findings described in their manuscript to be freely available to other researchers, either 1. In a public repository, 2. Within the manuscript itself, or 3. Uploaded as supplementary information.
This policy applies to all data except where public deposition would breach compliance with the protocol approved by your research ethics board. If your data cannot be made publicly available for ethical or legal reasons (e.g., public availability would compromise patient privacy), please explain your reasons on resubmission and your exemption request will be escalated for approval.

***> In addition to arching the data with Louisiana's Coastal Protection And Restoration Authority's public database, we have uploaded the data directly to the online data repository, Pangea.de. This should now comply with PLOS One's data policy.***

6. When completing the data availability statement of the submission form, you indicated that you will make your data available on acceptance. We strongly recommend all authors decide on a data sharing plan before acceptance, as the process can be lengthy and hold up publication timelines. Please note that, though access restrictions are acceptable now, your entire data will need to be made freely accessible if your manuscript is accepted for publication. This policy applies to all data except where public deposition would breach compliance with the protocol approved by your research ethics board. If you are unable to adhere to our open data policy, please kindly revise your statement to explain your reasoning and we will seek the editor's input on an exemption. Please be assured that, once you have provided your new statement, the assessment of your exemption will not hold up the peer review process.

***> We are now uploading the data directly to the online data repository, Pangea.de. This should now comply with PLOS One's data policy.***

7. Please include your full ethics statement in the ‘Methods’ section of your manuscript file. In your statement, please include the full name of the IRB or ethics committee who approved or waived your study, as well as whether or not you obtained informed written or verbal consent. If consent was waived for your study, please include this information in your statement as well.

> *Thank you for this comment. The manuscript now has the following section in the methods section: 3.10 Ethics Statement. Since the project did not involve the use of human subjects the use of an Institutional Review Board (IRD) was waved. Since the project did not involve the use of vertebrate or cephalopod animals, the use of an Institutional Animal Care and Use Committee (IACUC) or equivalent ethics committee was waved.*

8. We note that Figures 1-4, 6-10, 12,15 and 16 in your submission contain map/satellite images which may be copyrighted. All PLOS content is published under the Creative Commons Attribution License (CC BY 4.0), which means that the manuscript, images, and Supporting Information files will be freely available online, and any third party is permitted to access, download, copy, distribute, and use these materials in any way, even commercially, with proper attribution. For these reasons, we cannot publish previously copyrighted maps or satellite images created using proprietary data, such as Google software (Google Maps, Street View, and Earth). For more information, see our copyright guidelines: <http://journals.plos.org/plosone/s/licenses-and-copyright>.
 
We require you to either (a) present written permission from the copyright holder to publish these figures specifically under the CC BY 4.0 license, or (b) remove the figures from your submission:

We thank the editor for this important comment. Some of these figures indeed needed to be remade, while other of these figures were derived from publicly available sources such as the ones that are described by you. Here is a complete list of the figures, the status of their source material, and our actions.

*Figure 1. The top image comes from the US National Space and Aeronautics Administration, which makes its images freely available. The bottom image comes from the Europe Space Agency's Copernicus Sentinel Data and Service Information. This information is noted in the figure caption, and the public access statements for both NASA and Copernicus Sentinel have been uploaded.*

*Figure 2. This figure has been remade. The Google Earth images have been removed and replaced with images from the European Union's Copernicus Sentinel-2 Data. The public access statement for Copernicus Sentinel data has been uploaded.*

*Figure 3. This figure has been remade using images from the European Union's Copernicus Sentinel-2 Data. The public access statement for Copernicus Sentinel data has been uploaded.*

*Figure 4. This image comes from the Copernicus Sentinel Data Service, which makes its data publicly available. The public availability data statement from Copernicus has been uploaded.*

*Figure 5. No action was needed.*

*Figure 6. The background image comes from the Copernicus Sentinel Data Service, which makes its data publicly available. The public availability data statement from Copernicus has been uploaded.*

*Figure 7. This image comes from RCOAST, for which is run by co-author Christy Swann. We do not believe there is any proprietary information here.*

*Figure 8. The background image comes from the Copernicus Sentinel Data Service, which makes its data publicly available. The public availability data statement from Copernicus has been uploaded.*

*Figure 9. The background image comes from the Copernicus Sentinel Data Service, which makes its data publicly available. The public availability data statement from Copernicus has been uploaded.*

*Figure 10. The background image comes from the Copernicus Sentinel Data Service, which makes its data publicly available. The public availability data statement from Copernicus has been uploaded.*

*Figure 11: No was action needed.*

*Figure 12. The background image comes from the Copernicus Sentinel Data Service, which makes its data publicly available. The public availability data statement from Copernicus has been uploaded.*

*Figure 14. This figure has been replaced with a redrafted image. The original source is now referenced in the figure caption.*

*Figure 15. The top panels were replaced with redrafted images which are now cited in the figure caption.*

*Figure 16. The background image comes from the Copernicus Sentinel Data Service, which makes its data publicly available. The public availability data statement from Copernicus has been uploaded.*

9. Please include captions for your Supporting Information files at the end of your manuscript, and update any in-text citations to match accordingly. Please see our Supporting Information guidelines for more information: <http://journals.plos.org/plosone/s/supporting-information>.
