# Supplementary material for: Distributary development in a 21st century river: The evolution of Neptune Pass and its delta, the largest new offshoot of the Mississippi River
Source: PLoS One. 2025 Apr 16;20(4):e0320502. doi: 10.1371/journal.pone.0320502 (PMC12002485; doi:10.1371/journal.pone.0320502)
Supplement: Attachment — Submitted filename: Response_To_Reviewers_Feb_3.docx [file pone.0320502.s004.docx]

We thank the reviewer for these comments. They were helpful, and we appreciate the reviewer's comments. Please see our detailed responses below.

•Delta and delta lobe: The authors systematically refer to “delta” development with regards to the sediment accumulation associated with Neptune Pass. This is somewhat unfortunate as the background context is the Mississippi river and its DELTA. It may be less confusing to refer to delta LOBES (which all these other components of the Mississippi DELTA are), rather than individual DELTAS, e.g., lines 96-98, 121, 425-6. Sub-delta (line 699) is another new addition that does not clarify the picture.

*The reviewers' point is well taken. In this version, we use the term, "delta," when we speak of the generic geological feature. We use a, "D," when we refer to a specific named feature, such as the Mississippi River Delta." We have removed the term subdelta because we recognize that it could be confusing in some situations. See for example lines 96, 101, 769, 954.*

• Lines 41-42: “observation, coincides with an era when rivers have been controlled by large engineering projects”. Delete comma after observation and be less sweeping regarding river modification: “many rivers have been controlled ...”

*This has been completed. Please see line 41 of the track changes version*.

• Lines 48-49: “it is building is comprised .. Awkward statement.

This statement has been revised. Please see line 48 of the tracked changes version.

• Line 66: “..and often serve as a locus for human activities”. Use a more recent and more pertinent reference by some of the same authors:

- Anthony, E., Syvitski, J., Zăinescu, F., Nicholls, R.J., Cohen, K.M., Marriner, N., Saito, Y., Day, J., Minderhoud, P.S.J., Amorosi, A., Chen, Z., Morhange, C., Tamura, T., Vespremeanu-Stroe, A., Besset, M., Sabatier, F., Kaniewski, D., Maselli, V., 2024. Delta sustainability from the Holocene to the Anthropocene and envisioning the future. Nature Sustainability, 7,1235-1246. https://doi.org/10.1038/s41893-024-01426-3

*This reference has been updated as per the reviewer's helpful suggestion. Please see lines 1196-1199 of the tracked changes version.*

• Line 69: and alter the biogeochemistry and ecology of coastal waters [7–9]. Consider also the recent paper on tipping points in delta channels:

- van de Vijsel, R.C., Scheffer, M., Hoitink, A.J.F., 2024. Tipping points in river deltas. Nature Reviews Earth & Environment, 5, 843–858. https://doi.org/10.1038/s43017-024-00610-5

*This was a helpful suggestion, we appreciate hearing about this new paper. It is indeed quite interesting. However, we believe the current list of citations is sufficient.*

• Line 131-2: “It should be noted that these hypotheses are not mutually exclusive and represent end-members on a spectrum of sediment input possibilities”. Remove parentheses as this is an important point.

*This has been addressed. Please see lines 140-142 of the tracked changes version.*

• Line 173: “It is across the river ..”. Quaint or colloquial expression. Please rectify.

*This has been addressed. Please see lines 186-187 of the tracked changes version.*

• Line 223: “by placing ‘a’ rock wall ..”

*This has been addressed. Please see line 239 of the tracked changes version.*

• Line 225: “to inform comments on such this proposal …” Rectify.

*This has been addressed. Please see lines 241-242 of the tracked changes version.*

• Line 243: “and its potential impacts ‘on’… (on, in lieu of to).

*This has been addressed. Please see line 267 of the tracked changes version.*

• Lines 251-2: “data on discharge in the Mississippi River using data from multiple outside sources”. Awkward. Reword as “data on discharge in the Mississippi River and from multiple outside sources”

*This has been addressed. Please see lines 275-276 of the tracked changes version.*

• Line 255: “data was compiled”. Use rather “data were compiled”. Check manuscript and homogenize, as, sometimes, “data” is/are referred to in the singular (in abstract for instance : “there is relatively little data …) or plural form (line 149: “data that provide..”; line 290: “survey data were integrated ..”) form.

*This was addressed. Please see lines 279 and 309 of the tracked changes version.*

• Line 1069: Syvitski J, Anthony E, F. SYZ,. Check author name SYZ….

*This was addressed, the earlier version was missing a name. Please see lines 1196-1199 of the tracked changes version.*
